# Pancreatogastrostomy versus Pancreatojejunostomy: An Up-to-Date Meta-Analysis of RCTs

**DOI:** 10.1155/2017/7526494

**Published:** 2017-07-17

**Authors:** Konstantinos Perivoliotis, Eleni Sioka, Athina Tatsioni, Ioannis Stefanidis, Elias Zintzaras, Dimitrios Zacharoulis

**Affiliations:** ^1^Department of Surgery, University Hospital of Larissa, Mezourlo, 41110 Larissa, Greece; ^2^Postgraduate Programme (MSc): Research Methodology in Biomedicine, Biostatistics and Clinical Bioinformatics, University of Thessaly, Larissa, Greece; ^3^Research Unit for General Medicine and Primary Health Care, Faculty of Medicine, School for Health Sciences, University of Ioannina, Ioannina, Greece; ^4^Tufts University School of Medicine, Boston, MA, USA; ^5^Department of Nephrology, Medical School, University of Thessaly, Larissa, Greece; ^6^Department of Biomathematics, University of Thessaly School of Medicine, Larissa, Greece

## Abstract

**Background:**

A meta-analysis was conducted in order to provide an up-to-date comparison of pancreatogastrostomy (PG) and pancreatojejunostomy (PJ), after pancreatoduodenectomy (PD), in terms of clinically significant postoperative pancreatic fistula (POPF) and other postoperative complications.

**Methods:**

This meta-analysis was conducted according to the PRISMA guidelines and the Cochrane Handbook for Systematic Reviews of Interventions. A systematic literature search in MEDLINE and Cochrane Central Register of Controlled Clinical Trials was performed. Fixed Effects or Random Effects model was used, based on the Cochran *Q* test.

**Results:**

In total, 10 studies (1629 patients) were included. There was no statistical significance between PG and PJ regarding the rate of clinically significant POPF (OR: 0.70, 95%CI: 0.46–1.06). PG was associated with a higher rate of postpancreatoduodenectomy haemorrhage (PPH) (OR: 1.52, 95%CI: 1.08–2.14). There was no difference between the two techniques in terms of clinically significant PPH (OR: 1.35, 95%CI: 0.95–1.93) and clinically significant postoperative delayed gastric emptying (DGE) (OR: 0.98, 95%CI: 0.59–1.63).

**Discussion:**

There is no difference between the two anastomotic techniques regarding the rate of clinically significant POPF. Given several limitations, more large scale high quality RCTs are required.

## 1. Introduction

### 1.1. Rationale

Pancreatoduodenectomy (PD) is still the gold standard of treatment for patients with resectable benign and malignant lesions of the head of the pancreas and the periampullary region. Although PD is considered a safe operative technique, with 30-day mortality rates in specialized, high volume centers currently estimated below 3% [1, 2], complications, such as postoperative pancreatic fistula (POPF), delayed gastric emptying (DGE), and postpancreatoduodenectomy haemorrhage (POPH), increase the overall morbidity to the rate of 45%, despite the application of enhanced recovery approaches after surgery [3].

Given the fact that the frequency of POPF, the most notorious postpancreatoduodenectomy complication, remains as high as 40% [4], researchers have focused on factors that may influence this rate, with the pancreatoenteric anastomosis being one of them. The anastomosis between the pancreatic stump and the GI is regarded as prone to leakage, due to exposure of the suture line to pancreatic juice. The two most widely adopted postpancreaticoduodenectomy anastomotic techniques are the pancreatogastrostomy (PG) and the pancreatojejunostomy (PJ), which combined with anastomotic reinforcing techniques, such as glue and intraductal stenting, are designed to provide a sealed and stable pancreatoenteric junction. In the current literature, a series of retrospective and prospective studies [5–10] have compared PG and PJ with inconclusive results. Keck et al. [11], in a large multicenter randomized controlled trial, reported no difference between the two techniques in terms of clinically significant POPF, which is in contrast with results from previous meta-analyses [12–14], where it was suggested that PG was a safer and more effective method of reconstruction, with lower rates of POPF and other intra-abdominal complications and shorter length of hospital stay (LOS).

### 1.2. Objectives

In light of these conflicting evidences, we conducted a meta-analysis, in order to provide an up-to-date comparison of PG and PJ after PD, for benign or malignant diseases of the head of the pancreas and the periampullary region, in terms of clinically significant POPF and other postoperative complications.

## 2. Methods

### 2.1. Study Protocol

The conduction of this meta-analysis was completed according to the PRISMA [15] guidelines and the Cochrane Handbook for Systematic Reviews of Interventions. The present study was not registered in any database.

### 2.2. Primary Endpoint

The primary endpoint of this study was the rate of clinically significant postoperative pancreatic fistula (grade B/C according to ISGPF). POPF was defined by ISGPF [16] as a drain output of any measurable volume of fluid on or after POD 3 with an amylase content > 3 times the serum amylase activity. Classification to grades A, B, and C is based on the impact of POPF on the overall clinical course.

### 2.3. Secondary Endpoints

Secondary endpoints included overall postoperative POPF, postoperative delayed gastric emptying (DGE) [17], clinically significant DGE (grade B/C), postpancreatectomy haemorrhage (PPH) [18], clinically significant PPH (grade B/C), biliary fistula, intra-abdominal fluid collection, overall morbidity, mortality, reoperation rate, wound infection, intraoperative blood transfusion, operative time, and the length of hospital stay (LOS).

### 2.4. Eligibility Criteria

Eligible trials were prospective human studies with a RCT design, comparing PG and PJ after PD for benign or malignant diseases of the head of the pancreas and the periampullary region, whose outcome data were reported in English and could be retrieved. Excluded studies included those not written in English or studies with no outcome of interest and no comparison group and observational, nonhuman, or nonrandomized studies. Moreover, studies reported in the form of editorials, letters, conference abstracts, expert opinion, or duplicate studies were excluded.

### 2.5. Literature Search

A systematic literature search in electronic databases (MEDLINE and Cochrane Central Register of Controlled Clinical Trials) was performed (search date: 20 July 2016) in order to identify the eligible RCTs.

In order to perform the literature search the following keywords were used:

- *MEDLINE*: (Pancreaticoduodenectomy OR Pancreatoduodenectomy OR Whipple OR “pancreatoduodenal resection” OR “pancreaticoduodenal resection” OR pancreaticojejunostomy OR pancreatojejunostomy OR “pancreaticoenteric anastomosis” OR “pancreatoenteric anastomosis” OR pancreaticogastrostomy OR pancreatogastrostomy OR “pancreaticogastric anastomosis” OR “pancreatogastric anastomosis” OR “pancreaticojejunal anastomosis” OR “pancreatojejunal anastomosis”) AND (“Clinical Trials as Topic” OR “randomized controlled trial” OR “controlled clinical trial” OR randomized OR placebo OR randomly OR trial)
- *Cochrane Central Register of Controlled Clinical Trials (Wiley)*: (Pancreaticoduodenectomy OR Pancreatoduodenectomy OR Whipple OR “pancreatoduodenal resection” OR “pancreaticoduodenal resection” OR pancreaticojejunostomy OR pancreatojejunostomy OR “pancreaticoenteric anastomosis” OR “pancreatoenteric anastomosis” OR pancreaticogastrostomy OR pancreatogastrostomy OR “pancreaticogastric anastomosis” OR “pancreatogastric anastomosis” OR “pancreaticojejunal anastomosis” OR “pancreatojejunal anastomosis”)

### 2.6. Study Selection and Data Collection

After duplicate removal, titles and abstracts of the studies were screened according to eligibility criteria. The next step included the full text review of the articles in order to assess that they are consistent with the inclusion criteria.

All electronic database search, study selection, data extraction, and methodological assessment of the studies were performed blindly and in duplicate by two independent investigators (PK and SE). Disagreements were resolved by mutual revision and discussion, in order to reach a consensus. In case of not resolving the discrepancies, the opinion of a third investigator (TA) was considered.

From all eligible studies, the data extracted included author's name, study location and year, RCT type, sample size, the age and gender of the participants, primary outcome, follow-up duration, overall morbidity, underlying disease, operation type, rate of PD/pylorus preserving PD (PPPD), anastomotic technique, operative time, postoperative hospital stay, use of intraductal stent, glue and drains, postoperative administration of somatostatin, and information regarding the diameter of pancreatic duct and the texture of pancreas. Only results reported in the article of the studies were extracted.

All studies imported in this meta-analysis were submitted to rigorous quality and methodological evaluation for bias appraisal according to Cochrane's risk of bias assessing tool [19]. Validity checkpoints included assessment of random sequence allocation, allocation concealment, blinding of participants and personnel and blinding of outcome assessment, incomplete outcome data, and selective reporting. Cohen's *k* statistic was also calculated.

### 2.7. Statistical Analysis

Data analysis was performed using the Cochrane Collaboration RevMan version 5.3. Dichotomous variables were reported in the form of Odds Ratio (OR), while for continuous variables Weighted Mean Differences (WMD) were used. Results of the analyses were presented with the corresponding 95% Confidence Interval (95% CI).

In the case of continuous variables, if the article did not provide the mean and the Standard Deviation (SD), these were calculated from the median and the Interquartile Range (IR), based on the formula by Hozo et al. [20]. To be more specific, if the sample size was >25, then the mean was considered equal to the median. For sample sizes <70, SD was regarded as IR/4. If the sample size was >70, then SD was equal to IR/6. For dichotomous variables, the statistical method used was the Mantel-Haenszel (MH) and for continuous variables the Inverse Variance (IV). Both Fixed Effects (FE) and Random Effects (RE) model were calculated and reported. The decision of which model to finally estimate was based on the Cochran *Q* test. If statistically significant heterogeneity was present (*Q* test *P* < 0.1), then RE model was applied. Moreover, heterogeneity was quantified with the use of *I*^2^. The studies were weighted on the basis of sample size. Statistical significance was considered at the level of *P* < 0.05.

### 2.8. Risk of Bias across Studies

The funnel plot of the primary outcome was also visually inspected, in order to determine the possible presence of publication bias. An Egger's test was also performed for the primary outcome.

## 3. Results

### 3.1. Study Selection

**Figure 1:**
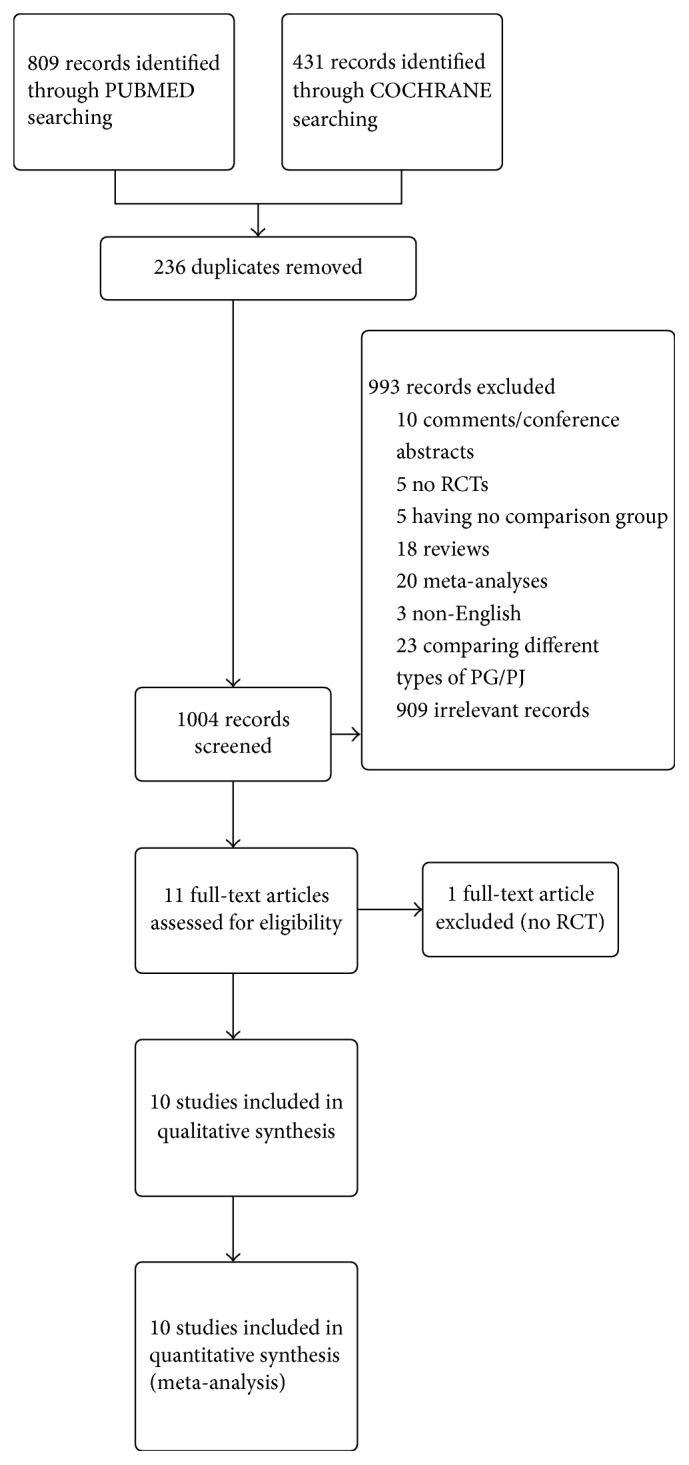
Study flow diagram.

From the literature search, 1240 citations (Figure 1) were retrieved, published up to 20 July 2016. After the removing of 236 duplicate records, the screening of the titles and the abstracts begun. From the 1004 studies submitted to the first phase of the screening, 993 were excluded. More specific, 10 were comments or conference abstracts, 5 did not have a RCT design, 5 did not have a comparison group, 18 were reviews of the current literature, 20 were meta-analysis, 3 articles were not written in English, 23 compared different techniques of PG or PJ instead, and 909 were irrelevant to the subject records. In full text review, 11 articles were submitted [9, 11, 21–29]. At this step, 1 trial [9] was rejected due to a no RCT design. Finally, 10 studies [11, 21–29] were included in qualitative and quantitative analysis.

### 3.2. Study Characteristics

Table 1 summarizes the characteristics of the included studies. The publication date ranges from 1995 up to 2016. Four studies were multicentered while the other six were single-centered. Fernàndez-Cruz et al. [24] were the first to adopt the ISGPS definition and classification of POPF. Since then, heterogeneity existed in the definition and diagnosis of POPF. The overall amount of patients included in this meta-analysis is 1629 (Table 2). A total of 826 PGs and 803 PJs were performed. The age of the participants extended from 12 to 87 years. Regarding the gender allocation between the two comparison groups, data are shown in Table 2. El Nakeeb et al. [23] compared the results of PG and an isolated Roux loop pancreatojejunostomy while Fernàndez-Cruz et al. [24], respectively, compared PJ and PG with gastric partition. In the rest of the studies, PG was considered the intervention and PJ the control. All studies, except Duffas et al. [22], had the rate of POPF as primary outcome. Four studies [21, 24, 26, 29] did not report the duration of follow-up. In the other six studies, follow-up varied from 30 days to 12 months. Regarding the underlying disease, carcinoma of the pancreatic head was the most frequent (Table 3). The PD and PPPD ratio is shown in Table 3. There was a lack of uniformity between the studies regarding the technique of PG and PJ anastomoses. Both PG and PJ could be performed in either a telescoped or a duct-to-mucosa manner.  Table 4 reports a summary of the studies implementing the use of stents in the pancreatic duct, anastomotic glue reinforcement, and the overall drain use. Postoperative octreotide was administered in 7 studies [21–23, 25–28]. All studies reported data regarding the main pancreatic duct diameter. Similarly, only Topal et al. [27] did not provide the allocation of the patients regarding pancreatic texture.

### 3.3. Risk of Bias within Studies

**Figure 2:**
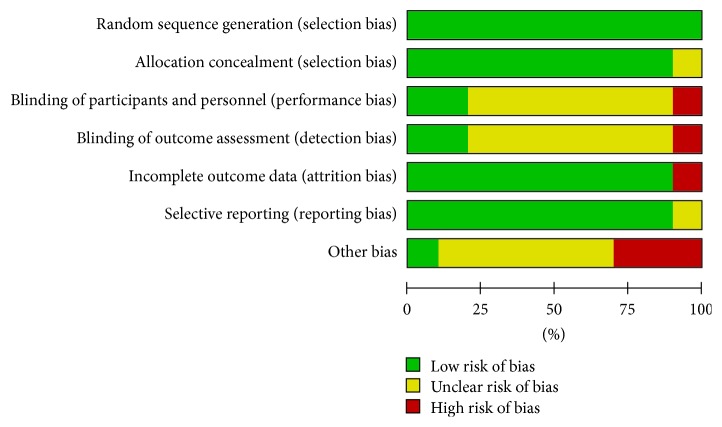
Risk of bias graph: review authors' judgments about each risk of bias item presented as percentages across all included studies.

**Figure 3:**
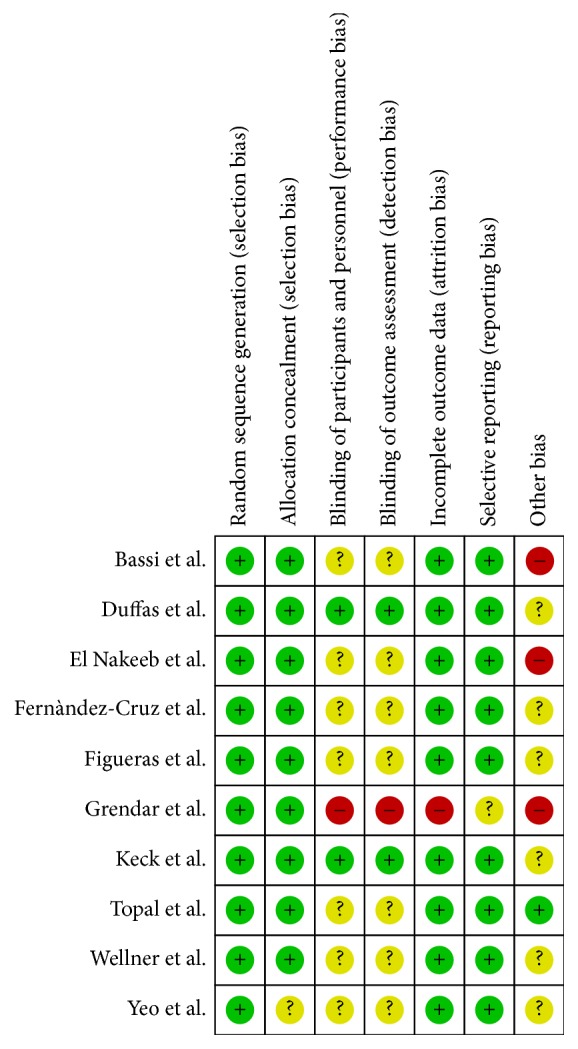
Risk of bias summary: review authors' judgments about each risk of bias item for each included study.

Figure 2 represents a summary of the included studies quality assessment. More specifically, as shown in Figure 3, all studies included a random sequence generation procedure in their protocol. Allocation concealment was also applied in all studies except one [29]. Only two trials [11, 22] reported the blinding of participants and personnel and the blinding of outcome assessment. Only in the study of Grendar et al. [26], incomplete outcome data and possible selective reporting were detected. There was almost perfect agreement between the two investigators (Cohen's *k* statistic: 82.3%  *P* < 0.001).

### 3.4. Primary Endpoint

**Figure 4:**
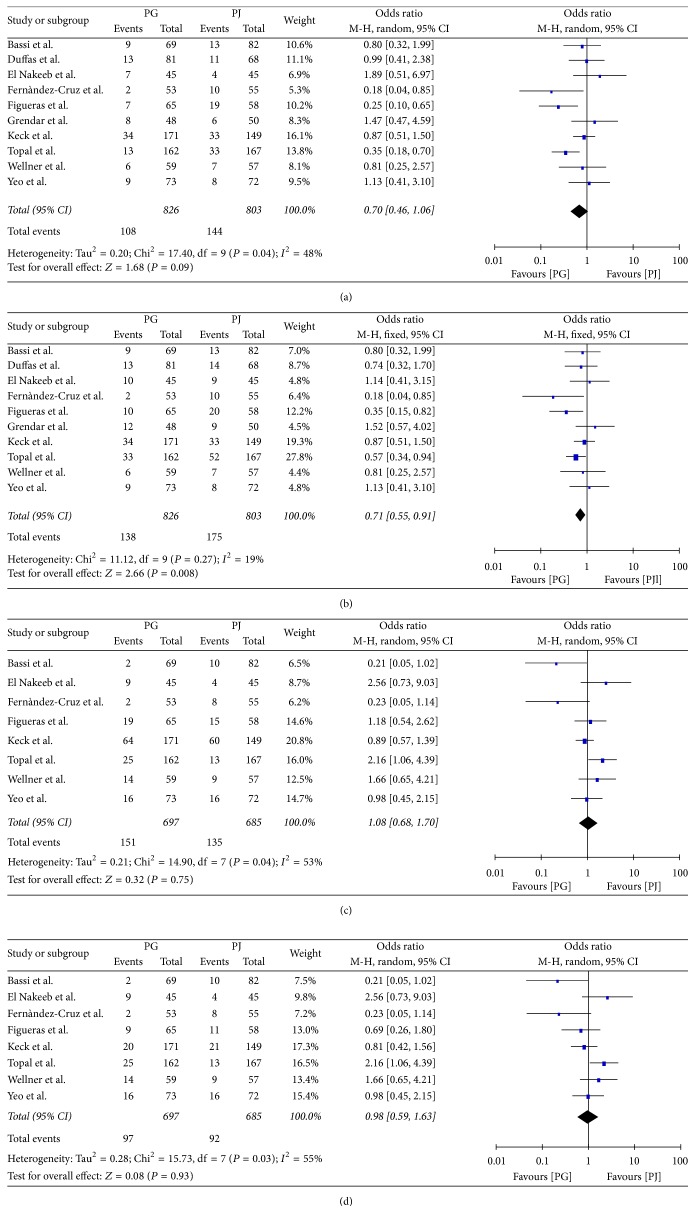
(a) Clinically significant postoperative pancreatic fistula, (b) postoperative pancreatic fistula, (c) delayed gastric emptying, and (d) clinically significant delayed gastric emptying.

- All ten studies (Figure 4(a)) compared the two anastomotic techniques regarding the clinically significant POPF. More specifically, 108 patients from a total of 826 in the PG group developed clinically significant POPF, whereas in the PJ group the same ratio was 144/803. Meta-analysis of these data showed no statistically significant (*P* = 0.09) difference between the two groups regarding clinically significant POPF (OR: 0.70, 95% CI: 0.46–1.06). Since there was significant heterogeneity between the studies (*Q* test *P*: 0.04, *I*^2^: 48% (95% CI: 0–75%)), a RE model was applied. Estimation of FE model did not wield consistent results (OR: 0.68, 95% CI: 0.51–0.89) with the RE model.

### 3.5. Secondary Endpoints

- All the included studies (Figure 4(b)) provided comparison between the two anastomotic techniques regarding POPF. In summary, 138 patients from a total of 826 submitted to PG developed POPF, instead of 175 and 803, respectively, in the PJ group. Meta-analysis of these data showed a statistically significant (*P* = 0.008) lower ratio of POPF (OR: 0.71, 95% CI: 0.55–0.91) for the PG group. Since there was no significant heterogeneity between the studies (*Q* test *P*: 0.27, *I*^2^: 19% (95% CI: 0–59.8%)), a FE model was applied. Estimation of RE model wielded consistent results (OR: 0.73, 95% CI: 0.54–0.98) with the FE model.
- Eight studies (Figure 4(c)) provided data for DGE. Meta-analysis of the data showed no statistically significant (*P* = 0.75) difference between the two groups regarding DGE (OR: 1.08, 95% CI: 0.68–1.70). Heterogeneity was significant between the studies (*Q* test *P*: 0.04, *I*^2^: 53% (95% CI: 0–78.9%)), so a RE model was used. Estimation of FE model wielded consistent results (OR: 1.07, 95% CI: 0.81–1.40) with the RE model.
- Eight studies (Figure 4(d)) provided data for clinically significant DGE. Meta-analysis of the data showed no statistically significant (*P* = 0.93) difference between the two groups regarding clinically significant DGE (OR: 0.98, 95% CI: 0.59–1.63). Heterogeneity was significant between the studies (*Q*test *P*: 0.03, *I*^2^: 55% (95% CI: 1.7%–79.8%)), so a RE model was used. Estimation of FE model wielded consistent results (OR: 1.03, 95% CI: 0.76–1.40) with the RE model.
- Eight studies (Figure 6(a)) provided data for PPH. Meta-analysis of the data showed statistically significant (*P* = 0.02) difference between the two groups regarding PPH (OR: 1.52, 95% CI: 1.08–2.14) in favor of PJ group. Heterogeneity was not significant between the studies (*Q* test *P*: 0.85, *I*^2^: 0% (95% CI: 0–80.3%)), so a FE model was used. Estimation of RE model wielded consistent results (OR: 1.52, 95% CI: 1.08–2.14) with the FE model.
- Eight studies (Figure 6(b)) provided data for clinically significant PPH. Meta-analysis of the data showed no statistically significant (*P* = 0.10) difference between the two groups regarding clinically significant PPH (OR: 1.35, 95% CI: 0.95–1.93). Heterogeneity was not significant between the studies (*Q* test *P*: 0.96, *I*^2^: 0% (95% CI: 0–75.9%)), so a FE model was used. Estimation of RE model wielded consistent results (OR: 1.35, 95% CI: 0.94–1.94) with the FE model.
- Seven studies (Figure 6(c)) provided data for biliary fistula. Meta-analysis of the data showed no statistically significant (*P* = 0.08) difference between the two groups regarding biliary fistula (OR: 0.58, 95% CI: 0.31–1.06). Heterogeneity was not significant between the studies (*Q* test *P*: 0.14, *I*^2^: 38% (95% CI: 0–73.7%)), so a FE model was used. Estimation of RE model wielded consistent results (OR: 0.58, 95% CI: 0.23–1.48) with the FE model.
- Nine studies (Figure 6(d)) provided data for intra-abdominal fluid collection. Meta-analysis of the data showed no statistically significant (*P* = 0.06) difference between the two groups regarding intra-abdominal fluid collection (OR: 0.64, 95% CI: 0.40–1.02). Heterogeneity was significant between the studies (*Q* test *P*: 0.07, *I*^2^: 45% (95% CI: 0–74.6%)), so a RE model was used. Estimation of FE model wielded consistent results (OR: 0.64, 95% CI: 0.47–0.87) with the RE model.
- Eight studies (Figure 7(a)) provided data for morbidity. Meta-analysis of the data showed no statistically significant (*P* = 0.82) difference between the two groups regarding morbidity (OR: 0.97, 95% CI: 0.77–1.23). Heterogeneity was not significant between the studies (*Q* test *P*: 0.21, *I*^2^: 28% (95% CI: 0–67.5%)), so a FE model was used. Estimation of RE model wielded consistent results (OR: 0.97, 95% CI: 0.73–1.28) with the FE model.
- Ten studies (Figure 7(b)) provided data for mortality. Meta-analysis of the data showed no statistically significant (*P* = 0.94) difference between the two groups regarding mortality (OR: 0.98, 95% CI: 0.60–1.61). Heterogeneity was not significant between the studies (*Q* test *P*: 0.94, *I*^2^: 0% (95% CI: 0–76.8%)), so a FE model was used. Estimation of RE model wielded consistent results (OR: 0.99, 95% CI: 0.60–1.64) with the FE model.
- Eight studies (Figure 7(c)) provided data for reoperation rate. Meta-analysis of the data showed no statistically significant (*P* = 0.33) difference between the two groups regarding reoperation rate (OR: 0.84, 95% CI: 0.59–1.20). Heterogeneity was not significant between the studies (*Q* test *P*: 0.79, *I*^2^: 0% (95% CI: 0–83%)), so a FE model was used. Estimation of RE model wielded consistent results (OR: 0.83, 95% CI: 0.58–1.20) with the FE model.
- Four studies (Figure 7(d)) provided data for wound infection. Meta-analysis of the data showed no statistically significant (*P* = 0.77) difference between the two groups regarding wound infection (OR: 1.08, 95% CI: 0.66–1.76). Heterogeneity was not significant between the studies (*Q* test *P*: 0.86, *I*^2^: 0% (95% CI: 0–90%)), so a FE model was used. Estimation of RE model wielded consistent results (OR: 1.08, 95% CI: 0.66–1.76) with the FE model.
- Six studies (Figure 8(a)) provided data for blood transfusion. Meta-analysis of the data showed no statistically significant (*P* = 0.86) difference between the two groups regarding blood transfusion (OR: 1.03, 95% CI: 0.72–1.47). Heterogeneity was not significant between the studies (*Q* test *P*: 0.39, *I*^2^: 5% (95% CI: 0–91.4%)), so a FE model was used. Estimation of RE model wielded consistent results (OR: 1.04, 95% CI: 0.72–1.51) with the FE model.
- Ten studies (Figure 8(b)) provided data for operative time. Meta-analysis of the data showed no statistically significant (*P* = 0.41) difference between the two groups regarding operative time (MWD: −5.73, 95% CI: −19.3, 7.85). Heterogeneity was significant between the studies (*Q* test *P*: <0.001, *I*^2^: 97% (95% CI: 0–98.1%)), so a RE model was used. Estimation of FE model did not wield consistent results (MWD: −16, 95% CI: −17.24, −14.76) with the RE model.
- Ten studies (Figure 8(c)) provided data for LOS. Meta-analysis of the data showed no statistically significant (*P* = 0.33) difference between the two groups LOS (MWD: −0.74, 95% CI: −2.24, 0.76). Heterogeneity was significant between the studies (*Q* test *P*: <0.001, *I*^2^: 91% (95% CI: 0–94.6%)), so a RE model was used. Estimation of FE model wielded consistent results (MWD: −0.06, 95% CI: −0.35, 0.23) with the RE model.

### 3.6. Risk of Bias across Studies

**Figure 5:**
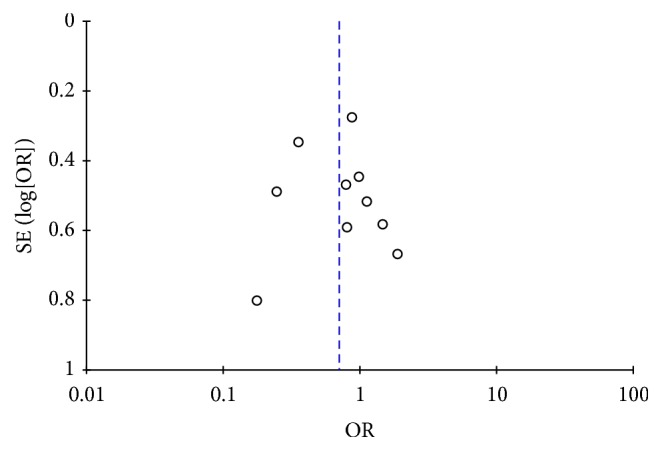
Funnel plot of comparison: postoperative pancreatic fistula.

**Figure 6:**
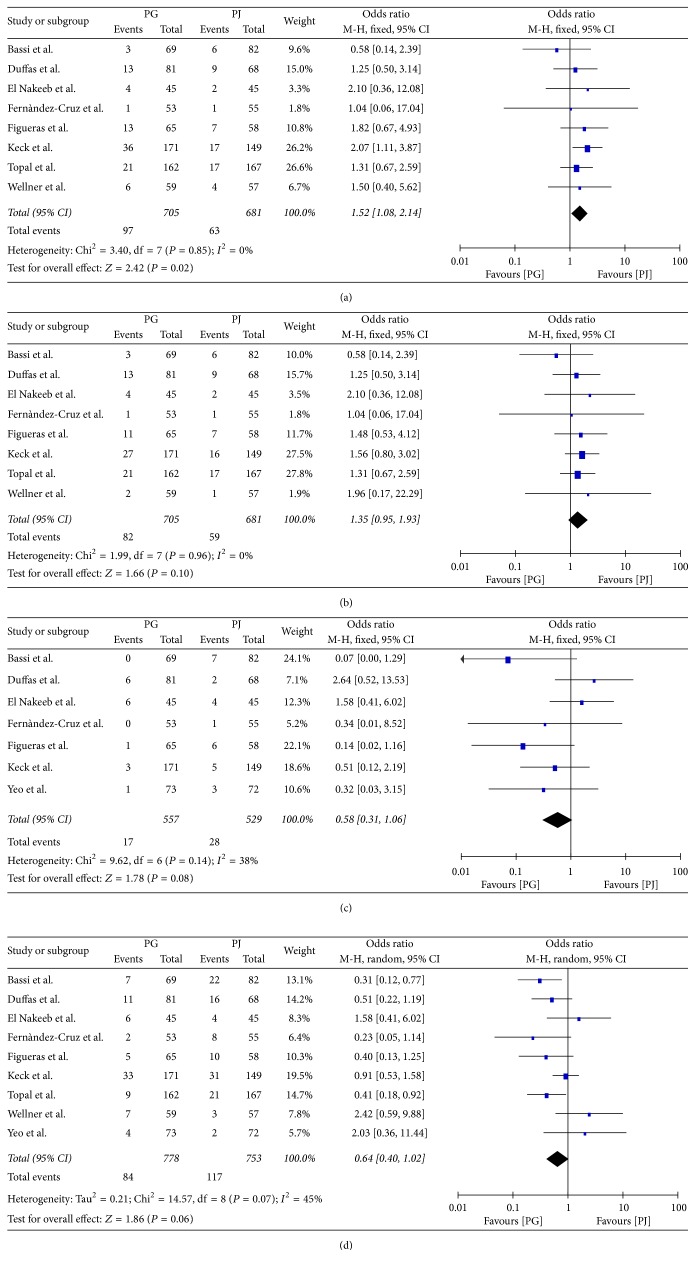
(a) Postpancreatectomy haemorrhage, (b) clinically significant postpancreatectomy haemorrhage, (c) biliary fistula, and (d) intra-abdominal fluid collection.

**Figure 7:**
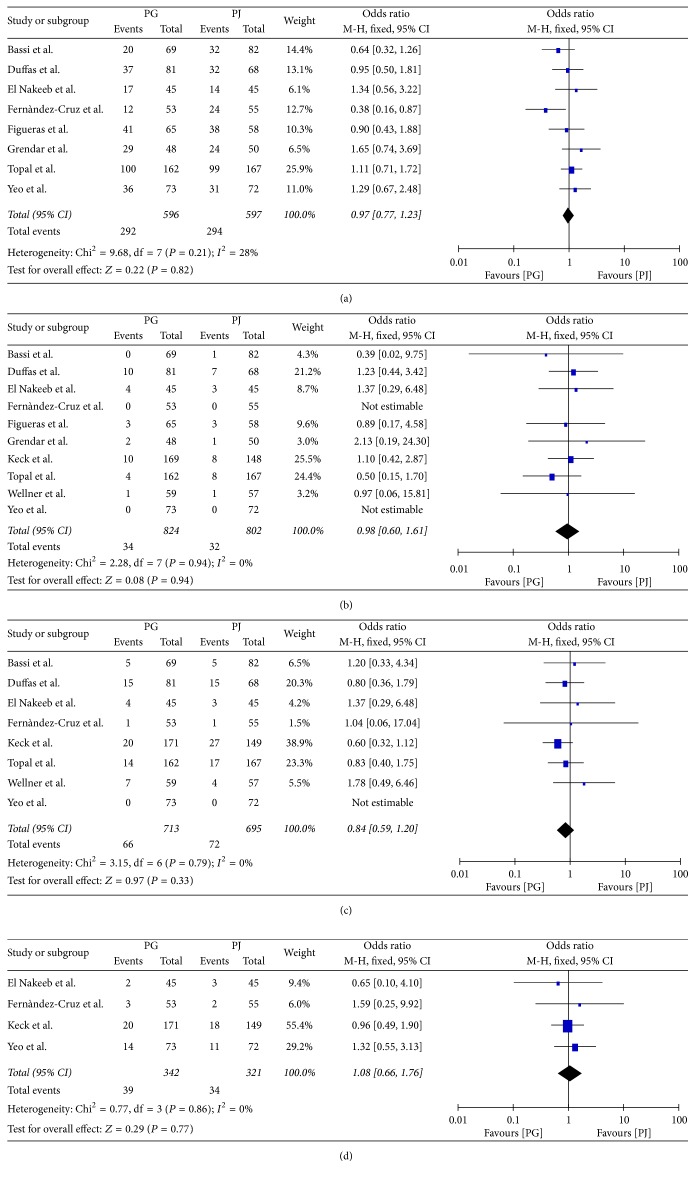
(a) Morbidity, (b) mortality, (c) reoperation, and (d) wound infection.

**Figure 8:**
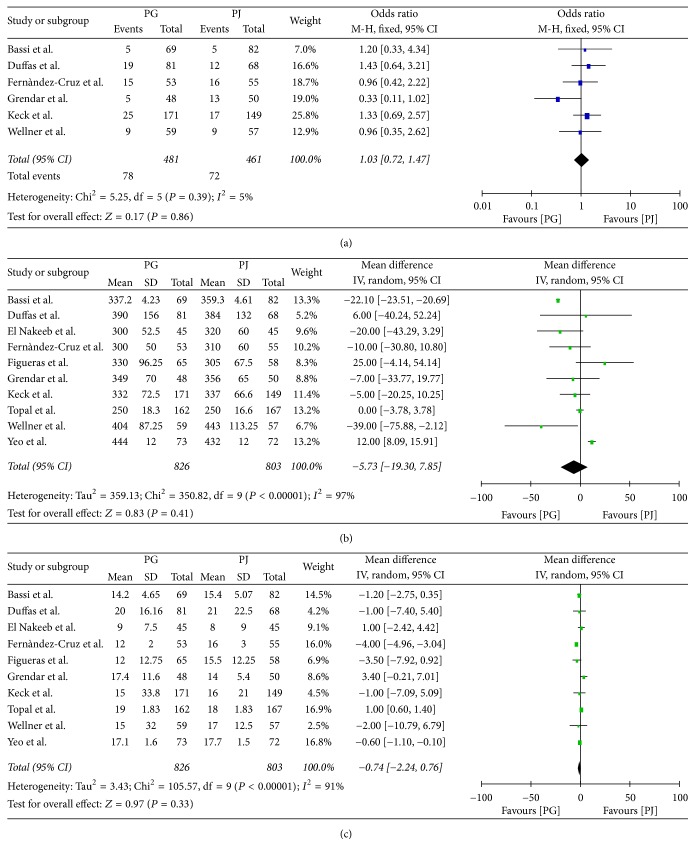
(a) Blood transfusion, (b) operative time, and (c) length of hospital stay.

Funnel plot of primary outcome (POPF) is shown in Figure 5. No study resides beyond the limits of 95% CI. Egger's test showed that there was no statistically significant publication bias (*P* = 0.951).

## 4. Discussion

### 4.1. Summary of Evidence

Pancreaticoduodenectomy remains the most widely used surgical modality for the treatment of pancreatic head and periampullary tumors. Failure of the pancreatic anastomosis resulting in POPF has been identified as one of the most important factors of postoperative morbidity. It must also be mentioned that POPF is assumed to have a close relationship with other post-PD complications, such as IAC, DGE, and PPH [30, 31]. As a result, surgeons, in an attempt to minimize post-PD complications have meticulously compared the available anastomotic techniques.

In our study, after a systematic literature search, a meta-analysis of available RCTs was performed. In the qualitative and quantitative analysis, 10 studies with a total of 1629 patients were included. Regarding the primary outcome, PG was not superior to PJ. However, this result was different when the two techniques were compared on the basis of overall POPF, where a significant difference was found. Heterogeneity in clinically significant POPF could possibly be the result of nonuniformity in the definition of POPF. Although the included studies after 2005 were consistent with the 2005 ISGPS POPF definition, the remaining defined POPF in an inconsistent way. DGE and clinically significant DGE were found to have no difference between PG and PJ, with a high level of heterogeneity though. As the operation type was not determined in most eligible studies, surgeons performed either PD or PPPD. The above-mentioned heterogeneity could be explained in the light of lack of stratification regarding the operation type.

Respectively, results from pooled data showed a lower rate of PPH for PJ, but no difference for clinically significant PPH. Heterogeneity for both of them was 0%, increasing thus the validity of these findings. The rate of biliary fistula and the intra-abdominal fluid collection was not significantly different between PG and PJ, which diverges from the results of previous studies [32–35], due to inclusion of the recent RCTs [11, 26]. Moreover, overall postoperative morbidity for both techniques was estimated at the level of 49%, complying with current literature [4]. Similarly, no difference was found in terms of mortality, reoperation rate, wound infection, and perioperative blood transfusion. Finally, PG was not superior to PJ in terms of operative time and LOS. Heterogeneity was significantly high in these comparisons, possibly due to the approximate calculation of the mean and SD.

Risk factors for development of POPF are the age, gender of the patient, preoperative jaundice and malnutrition, underlying pathology, cirrhotic liver, BMI, soft pancreas, pancreatic diameter, pancreatic duct size, operative time, resection type, anastomotic technique, and intraoperative blood loss [36]. El Nakeeb et al. [31], however, in a retrospective study of 471 patients, suggested that risk factors for POPF include the cirrhotic liver, BMI, soft pancreas, pancreatic diameter < 3 mm, and pancreatic duct near the posterior border.

The superiority of PG over PJ in terms of POPF can be justified by some theoretical advantages. Firstly, due to the fact that the posterior wall of the stomach lies just above the pancreatic remnant, the tension between the stomach and the pancreatic stump is minimized. Secondly, the acidic gastric content prevents the activation of pancreatic enzymes and consequently the anastomotic lysis. Moreover, compared to a jejunum loop, the stomach wall is thicker, thus stabilizing the anastomosis. Finally, the abundant stomach wall vascularization decreases the chance of an anastomotic ischaemia. This may also be the reason of increased post-PD PPH in the PG group, rendering perioperative meticulous haemostasis of utmost importance.

As far as postoperative exocrine pancreatic function is concerned, data are scarce and inconsistent, thus making further analysis very difficult. More specifically, a higher stool elastase level and a significant lesser weight loss were reported in the PG group [25]. Comparing PG and IRPJ, El Nakeeb et al. [23] concluded that postoperative steatorrhea and need for pancreatic enzyme supplements were higher in the PG group, while post-PD serum albumin was in a lower level in patients submitted to PG. On the contrary, the need for oral enzyme supplements, six months after surgery, was lower in the PG group, with the rate of reported steatorrhea further decreasing after 12 months [11]. In a study of 99 patients, Hirono et al. [37] identified hard texture of pancreas and PG reconstruction as individual risk factors for postoperative pancreatic exocrine function insufficiency.

Regarding the pancreatic endocrine function, El Nakeeb et al. [23] showed that, although there was no difference in the overall rate of postoperative diabetes mellitus between PG and IRPJ, postoperative fasting blood sugar was higher in the PG group. Furthermore, fasting blood sugar increased postoperatively in the PG group, unlike IRPJ, where fasting blood sugar was significantly lower after surgery. However, two studies claimed that there was no statistically significant difference between PG and PJ in the rate of de novo diabetes mellitus [11, 25].

Morphological outcomes were not systematically provided and therefore a pooled analysis could not be reported. Data show that pancreatic duct tended to be more dilated in the PG group, even after a median of 32 months and the pancreatic parenchyma density is significantly decreased [38, 39]. A significant higher impact of postoperative atrophy of the pancreatic parenchyma was recorded in the PG group [39]. However, in a study by Fang et al. [40], no significant differences between PG and PJ regarding postoperative pancreatic duct diameter were reported.

Our meta-analysis provides an up-to-date pooled, published only data, estimation of the rate of POPF, and other postoperative complications between the two most popular anastomotic techniques. Compared to other recent studies [12, 41], it reports results not only in overall morbidity but also in clinically significant complications, such as DGE and PPH.

### 4.2. Limitations

Several limitations should be taken into account before appraising the results of this meta-analysis. First of all, the between studies heterogeneity was substantial, limiting, in this way, the significance of the results. Furthermore, there is a diversity in the POPF definition among the included studies. It must be noted, though, that all studies after 2005 use the ISGPS definition. The included trials have also incorporated both PD and PPPD in their study groups and there was, also, no stratification on the basis of the underlying pathology. Moreover, a lack of uniformity exists, regarding the surgical anastomotic technique that may possibly result in biased results. Factors like the texture of pancreas and the pancreatic duct diameter might also influence the results. Another source of bias could be the perioperative use of glue and stents and the postoperative administration of somatostatin, since not all studies reported this information. Another factor that contributes to heterogeneity is the surgical experience in the applied anastomotic technique. Last literature search was performed 20 July 2016. The new refinement of the ISGPS POPF definition [42] was published later; thus, it was not incorporated.

### 4.3. Conclusions

The present meta-analysis of RCTs demonstrates that there is no difference between the two anastomotic techniques regarding clinically significant POPF. PG has lower overall incidence of POPF and higher rate of PPH against PJ. Moreover, PG and PJ did not differ in terms of overall DGE, clinically significant DGE, clinically significant PPH, biliary fistula, intra-abdominal fluid collection, overall morbidity, mortality, reoperation rate, wound infection, intraoperative blood transfusion, operative time, and LOS. Therefore, selection of proper pancreatic reconstruction should be according to the risk of patients, in order to reduce POPF, postoperative complications, and mortality. PG is superior to PJ regarding short term outcomes, while PJ provides better pancreatic function. Given several limitations, more large scale high quality RCTs are required for the effect of the anastomotic technique on the incidence of POPF to be clarified.

## Figures and Tables

**Table 1:** Included studies.

PMID	First author	Country	Publication year	RCT type	POPF definition
26135690	Keck	Germany	2016	Multicenter, randomized, controlled, observer- and patient-blinded trial	ISGPS (grade B/C)

25799130	Grendar	Canada	2015	Single-center, randomized, controlled trial	Radiologically proven anastomotic leak or continued drainage of lipase-rich fluid on PoD 10. Classification by ISGPS

24467711	El Nakeeb	Egypt	2014	Single-center, prospective, randomized study	ISGPS (grade A/B/C)

24264781	Figueras	Spain	2013	Multicenter, prospective, randomized study	ISGPS (grade B/C)

23643139	Topal	Belgium	2013	Multicenter, randomised, superiority trial	ISGPS (grade B/C)

22744638	Wellner	Germany	2012	Single-center, open, randomized, controlled study	ISGPS (grade B/C)

19092337	Fernàndez-Cruz	Spain	2008	Single-center, prospective, randomized study	ISGPS (grade B/C)

16327486	Bassi	Italy	2005	Single-center, prospective, randomized study	Any clinical significant output of fluid, rich in amylase, confirmed by fistulography

15910726	Duffas	France	2005	Multicenter, single blind, controlled, randomized trial	Fluid obtained through drains or percutaneous aspiration, containing at least 4 times normal serum values of amylase for 3 days or as anastomotic leaks shown by fistulography

7574936	Yeo	USA	1995	Single-center, prospective, randomized trial	Drainage of greater than 50 mL of amylase rich fluid (greater than threefold elevation above upper limit of normal in serum) through the operatively placed drains on or after

**Table 2:** Study characteristics.

First author	Sample size		Age		Gender (M/F)		Intervention	Comparator	Primary outcome	Follow-up	Morbidity	
	PG	PJ	PG	PJ	PG	PJ					PG	PJ
*Keck*	171	149	68 (35–86)	66 (29–87)	95/76	93/56	PG	PJ	Clinically relevant POPF, grade B or C	12 months	N/A	

*Grendar*	48	50	63.6 ± 13.1	68.1 ± 10.7	20/28	29/21	PG	PJ	Rate of pancreatic anastomotic leak/fistula	N/A	29	24

*El Nakeeb*	45	45	58 (12–73)	54 (15–73)	23/22	27/18	PG	Isolated Roux loop pancreaticojejunostomy	Rate of POPF	12 months	17	14

*Figueras*	65	58	67 (35–80)	65.5 (42–80)	44/21	37/21	PG	PJ	Rate of POPF	6 months	41	38

*Topal*	162	167	67.0 (60.6–73.5)	66.1 (59.4–74.6)	100/62	91/76	PG	PJ	Clinically relevant POPF, grade B or C	2 months	100	99

*Wellner*	59	57	67 (34–84)	64 (23–81)	27/32	29/28	PG	PJ	Clinically relevant POPF, grade B or C	90 days	N/A	

*Fernàndez-Cruz*	53	55	63 ± 13	63 ± 14	29/24	38/17	PG with gastric partition	PJ	Rate of POPF	N/A	12	24

*Bassi*	69	82	59.3 (58.2–60.4)	55.5 (54.5–56.6)	44/25	35/33	PG	PJ	Rate of POPF	N/A	20	32

*Duffas*	81	68	58.2 ± 11	58.6 ± 12	51/30	35/33	PG	PJ	Rate of one or more postoperative IACs	30 days	37	32

*Yeo*	73	72	61.5 ± 1.7	62.4 ± 1.4	33/40	38/34	PG	PJ	Rate of POPF	N/A	36	31

**Table 3:** Operative characteristics.

First author	Disease (PDAC/DD/AMP/DBD/OTHER)		Operation type	pd/pppd		Technique		Operative time		Postoperative hospital stay	
	PG	PJ		PG	PJ	PG	PJ	PG	PJ	PG	PJ
*Keck*	104/-/10/-/14	98/-/11/-/14	pd or pppd	37/134	28/121	Dunking, pursestring, or interrupted or combination suture	Duct to mucosa or dunking, running, or interrupted or combination suture	332 (165–600)	337 (165–565)	15 (5–208)	16 (3–129)

*Grendar*	N/A		pd or pppd	N/A		Posterior gastrostomy, 2-layer anastomosis	2-layer end-to-side anastomosis	349 ± 70	356 ± 65	17.4 ± 11.6	14.0 ± 5.4

*El Nakeeb*	26/2/17/0/0	20/4/19/2/0	pd	45/0	45/0	Posterior gastrostomy, 2-layer anastomosis	Two-layer end-to-side pancreaticojejunostomy	300 (210–420)	320 (240–480)	9 (4–34)	8 (5–41)

*Figueras*	33/6/8/8/10	29/10/7/3/19	pd or pppd	35/30	30/28	Posterior gastrostomy double-layer invaginated	Duct-to-mucosa pancreaticojejunostomy	330 (235–620)	305 (240–510)	12 (1–52)	15,5 (6–55)

*Topal*	98/11/23/28/2	107/14/28/15/3	pd or pppd	65/98	65/102	End-to-side telescoped antecolic posterior gastrostomy	End-to-side telescoped pancreaticojejunostomy	250 (210–320)	250 (210–310)	19 (14–25)	18 (14–25)

*Wellner*	26/3/9/2/8	30/2/7/2/10	pd or pppd	7/52	2/55	Invagination, posterior pancreatogastrostomy with pursestring suture	Duct-to-mucosa pancreaticojejunostomy	404 (280–629)	443 (230–683)	15 (7–135)	17 (10–60)

*Fernàndez-Cruz*	26/1/12/8/9	28/1/10/7/9	pppd	0/53	0/55	End-to-side duct-to- mucosa pancreatogastrostomy	End-to-side duct mucosa anastomosis PPPD-PJ	300 ± 50	310 ± 60	12 ±2	16 ± 3

*Bassi*	32/1/13/1/22	28/1/11/2/40	pd or pppd	3/66	12/70	Posterior single-layer telescoped gastrostomy	Single-layer pancreaticojejunal or duct to mucosa	337.2 (336.1–338.2)	359.3 (352.9–354.9)	14.2 (13.1–15.3)	15.4 (14.3–16.5)

*Duffas*	34/3/17/8/19	25/3/19/11/10	pd or pppd	63/18	50/18	Depending on surgeon's preference	Depending on surgeon's preference	6.5 ± 2.6 (h)	6.4 ± 2.2 (h)	20 (1–98)	21 (7–97)

*Yeo*	40/4/7/6/16	40/5/11/7/9	pd or pppd	13/60	13/59	Posterior gastrostomy	End-to-end or end-to-side pancreaticojejunostomy	7.4 ± 0.2 (h)	7.2 ± 0.2 (h)	17.1 ± 1.6	17.7 ± 1.5

**Table 4:** Intraoperative characteristics.

First author	Stent		Postoperative octreotide		Anastomotic glue reinforcement		Drains		Pancreatic parenchyma (soft/hard)		Pancreatic duct diameter	
	PG	PJ	PG	PJ	PG	PJ	PG	PJ	PG	PJ	PG	PJ
*Keck*	N/A		N/A		N/A		N/A		95/66	83/62	94 (<3 mm)	78
*Grendar*	10	39	42	39	N/A		38	44	25/23	18/32	3.8 ± 2.4 (mm)	4.3 ± 2.6
*El Nakeeb*	0	0	45	45	N/A		N/A		26/19	22/23	22 (<3 mm)	21
*Figueras*	N/A		65	58	N/A		65	58	34/31	33/25	4 (1–15) (mm)	4 (1–11)
*Topal*	0	0	162	167	0	0	162	167	N/A		98 (<3 mm)	102
*Wellner*	0	57	22	13	N/A		59	57	35/23	29/28	26 (<3 mm)	18
*Fernàndez-Cruz*	53	55	0	0	N/A		53	55	24/29	25/30	3.0 ± 1.7 (mm)	3.0 ± 1.6
*Bassi*	0	0	69	82	N/A		69	82	69/0	82/0	<5 mm	
*Duffas*	15	15	22	22	17	12	81	68	49/32	41/27	32 (<3 mm)	31
*Yeo*	0	0	0	0	0	0	73	72	16/21	17/28	3.4 ± 0.2 (mm)	2.9 ± 0.2
